# Unintentional injuries in the rural population of Twiserkan, Iran: A cross-sectional study on their incidence, characteristics and preventability

**DOI:** 10.1186/1471-2458-8-269

**Published:** 2008-07-31

**Authors:** Forouzan Rezapur-Shahkolai, Mohsen Naghavi, Mohammadreza Shokouhi, Lucie Laflamme

**Affiliations:** 1 Division of International Health, Department of Public Health Sciences, Karolinska Institutet, Stockholm, Sweden; 2 Hamadan University of Medical Sciences, Hamadan, Iran; 3 National Public Health Management Centre, Tabriz, Iran; 4 Institute for Health Metrics and Evaluation, Washington University, Seattle, USA

## Abstract

**Background:**

Knowledge is sparse concerning injuries affecting rural populations in low and middle-income countries in general and in Iran in particular. This study documents the incidence and characteristics of severe injuries affecting rural people in the Iranian district of Twiserkan and it investigates these people's suggestions for injury prevention and control.

**Methods:**

An interview-based investigation was undertaken that comprised all unintentional injuries leading to hospitalization (more than 6 hours) or death that had occurred within a twelve month period and that were identified in the files of the 62 "health houses" of the Twiserkan district. For each case, semi-structured interviews were conducted at the households of the injured people (134 injuries affecting 117 households were identified).

**Results:**

The incidence rates of fatal and non-fatal injuries were respectively 4.1 and 17.2 per 10 000 person-years and, as expected, men were more affected than women (77.6% of all injury cases). Traffic injuries (in particular among motorcyclists) were as common as home-related injuries but they were far more fatal. Among common suggestions for prevention, people mentioned that the authorities could work on the design and engineering of the infrastructure in and around the village, that the rural health workers could contribute more with local information and education and that the people themselves could consider behaving in a safer manner.

**Conclusion:**

Not only domestic injuries but also those in traffic are an important cause of severe and fatal injury among rural people. Health workers may play an important role in injury surveillance and in identifying context-relevant means of prevention that they or other actors may then implement.

## Background

Injuries constitute an important health problem worldwide and they are one of the major causes of death among people under 45 years old [1,2]. The majority of all injury-related deaths occur in low and middle-income countries [1-4] where knowledge is scarce regarding injury distribution, pattern and prevention [5]. Epidemiological studies have been conducted in some low and middle-income countries but, most often, traffic-related injuries and those occurring in urban settings have been in focus [6-8]. Yet, studies in rural areas have been conducted in countries in various continents, including Asia (Pakistan [9], Bangladesh [10,11], India [12], and Vietnam [5,13]), Africa (Kenya [14], Ghana [15], Uganda [16] and Tanzania [17]) and South America (Nicaragua [18]). Those studies reveal that injuries constitute an important health problem in the rural areas.

In Iran, where this research has been conducted, injury-related years of life lost are higher than for the worldwide average [19,20]. Studies on injury epidemiology and prevention are limited and are mainly urban [21-23]. Also people's experiences and opinions about injury prevention and control have rarely been addressed. Currently, approximately 33% of the total population live in rural areas [24], and people benefit from a well-established health network, consisting of village-based local "health houses", from which health workers (known as Behvarzes) work. The main function of the Behvarz is to offer primary health care services to the local population and to gather health information. Usually the Behvarzes are selected from their local community and can therefore establish a very close relationship with community members. This, in turn, can help to gather accurate data. Health house workers also contribute to the simple but well-integrated health information system [25].

In 2004, the Ministry of Health and Medical Education launched a program ultimately aiming at the reduction of injuries in rural areas. As a first step, an injury registration and surveillance system has been developed that forms part of the health information system and implies that the Behvarzes are responsible for the registration of all injuries leading to hospitalization (at least 6 hours as a standard criterion) or death. This data should provide information on the frequency of occurrence and characteristics of severe injuries and allow for the follow-up of future national and local interventions. Twiserkan district, where this study was conducted, is one of the districts selected for the pilot phase of the implementation of the surveillance system.

In the current study, we take advantage of the reports gathered by the Behvarzes over a one year period to assess the incidence of rural injuries and, through interviews with injured people or their relatives, characterize those injuries' epidemiology and document the suggestions of people from affected families concerning injury prevention and control.

## Methods

The Twiserkan district is located in the Hamadan province (over 19 000 square kilometers), in western Iran. In 2002, Hamadan had over 1.7 million inhabitants, of whom 44% lived in rural areas [26,27]. In 2006, when this study was conducted, Twiserkan district had a population of about 110 000 inhabitants, of which 58% was rural. The number of rural households was 14,789 and the number of inhabitants amounted to 62,857.

All unintentional injuries leading to hospitalization (more than 6 hours) or death, occurring over a one-year period (June 1, 2005 – May 31, 2006), were considered. These were first identified in the files compiled at the local health houses (n = 62) in the Twiserkan district (see below). Thereafter the household of each injured person was visited by a trained and experienced interviewer. Before any interview, the aim of the study and main content of the interview were explained to the interviewee who was also guaranteed confidentiality. Once verbal consent was given, a short face-to-face interview was conducted (in June 2006; 134 injuries in 117 households) with the family member identified as responsible of caring for the household (response rate 100%). To ensure as complete answers as possible, the injured family member took part in the interview any time she or he was present at the time of visit. This was the most common situation, except of course for fatal injuries. It can be underlined that the recall period ranged from a few days to one year post injury. About two-thirds of the injuries had occurred between 6 to 12 months before the interview and the remaining occurred either 3 to 6 (13.4%) or less than 3 months (18.7%).

Prior to data collection, a structured questionnaire including both closed and open questions was developed and pre-tested by the research team. It included information about both the injured person (and his/her household) and the injury (type, nature and circumstances of occurrence). Open-ended questions were included so as to find out how people regarded the role of the community, the Behvarzes, and the authorities (health and others) with regard to injury prevention. For each actor, people were asked to give their opinion as to what more could be done to help reduce injury for the village residents. In households with more than one injury during the reference period, all injuries sustained were considered at once when addressing the questions about household opinions concerning the roles of different actors in injury prevention and control.

The study was approved by the Iranian National Ethics Committee in Medical Research, Ministry of Health and Medical Education of Iran.

Data were entered, processed and analyzed in Excel (version 2003). Injury incidence rates were estimated globally and for fatal and non-fatal injuries respectively. Injury characteristics were coded and categorized according to the WHO guidelines for injury survey and surveillance [28,29]. Thereafter, using descriptive statistics, the characteristics of the injured people (sex, age group, education and occupation), of the injury events (place of injury and injury mechanism) and of their consequences (nature, body region and injury severity/recovery) were highlighted. When a person sustained several injuries during the same injurious event, the most severe one was considered. This was made possible as space was provided in the questionnaire to identify the most severe injury, following the WHO guidelines and after discussion and consensus between two members of the research team.

People's opinions about injury prevention were first entered as free text. Thereafter, answers were read by two members of the research team and key ideas/phrases were identified and discussed at different sessions. Meaningful categories were identified that represented specific and homogeneous domains of potential intervention or action. Since some people had several suggestions and as they were not asked to prioritize or rank them, all opinions expressed by each respondent were taken into account.

## Results

### Injury incidence of non-fatal and fatal injuries

A total of 134 injuries were reported by the Behvarzes during the study period. These were identified among 117 households (of 14,789 in total) and consisted of 26 fatal and 108 non-fatal injuries. The corresponding incidence rates of injuries per 10 000 person-years are therefore 21.4 injuries in total (95% CI 17.7–24.9), 4.1 fatal injuries per 10 000 person-years (95% CI 2.5–5.7) and 17.2 non-fatal injuries (95% CI 13.9–20.4).

Injury deaths occurred most often in traffic crashes (n = 10), followed by burns (n = 6), poisoning (n = 5), falls (n = 4), and electrocution (n = 1). Of the 134 injury cases identified, 22 were attributable to seven injurious events: three traffic-related and four in the home (see below). In the remainder of the text, all injured people, even those injured in the same event, are considered as individual cases.

### Injured people's characteristics

Table 1 presents the characteristics of the injured people. In total, about three-quarters were males; 21.6% were aged 15 or less and an additional 22.4%, 56 and over. The majority had not completed high school (85.8%) and 26.1% were farmers.

**Table 1:** Characteristics of the fatal and non-fatal unintentional injured people (June 1, 2005-May 31, 2006)

**Characteristics**	**Number**	**%**
**Sex**		
Male	104	77.6
Female	30	22.4
		
**Age group (in years)**		
< 1	1	0.7
1–5	8	6.0
6–15	20	14.9
16–25	25	18.7
26–35	17	12.7
36–45	17	12.7
46–55	16	11.9
56–65	13	9.7
66+	17	12.7
		
Education*		
High school graduate (high school is grades 9 to 12) or above	9	6.7
Secondary school graduate (secondary school is grades 6 to 8) and/or some high school	24	17.9
Completed primary school (primary school is up to grade 5) and/or some secondary school	34	25.4
Primary school not completed	20	14.9
No schooling	37	27.6
N/A (children under 6 years old)	10	7.5
		
**Occupation**		
Farmer	35	26.1
Other self employed	15	11.2
Student	24	17.9
Housewife	20	14.9
Unemployed	14	10.4
Labourer	13	9.7
Retired	10	7.5
Other (governmental employee and conscript)	3	2.2
N/A (children under 10 years old)	10	7.5

*In decreasing order according to the Iranian system.

### Fatal and non-fatal unintentional injury characteristics

**Figure 1:**
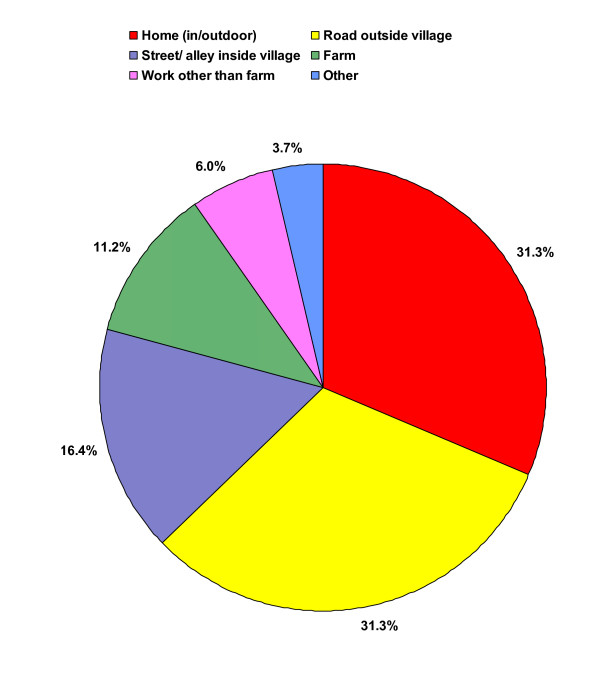
Places of occurrence of fatal and non-fatal unintentional injury events (June 1, 2005 – May 31, 2006).

**Figure 2:**
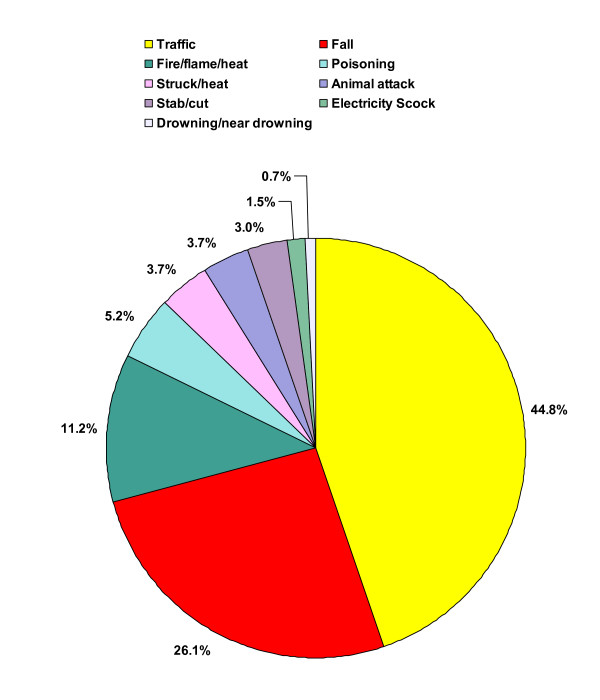
Mechanisms of fatal and non-fatal unintentional injuries (June 1, 2005 – May 31, 2006).

Figures 1 and Figure 2 show the characteristics of the fatal and non-fatal unintentional injury circumstances (place of injury and injury mechanism). The injuries occurred in similar proportions in the home or on the road, outside the village. Traffic injury was by far the most common injury mechanism (44.8%), followed by falls (26.1%) and thereafter burns (fire/flame/heat; 11.2%). More details of these three injury mechanisms are given in the text below.

#### Road Traffic Injuries (RTIs; 60 injuries from 56 households)

Of 60 RTIs, 48 were sustained by males and the bulk of them were among people of working age, most often 16–35 years (28 cases). Only 10 cases were reported among children 15 years and less. The most common mode of transportation among the injured people was by motorcycle (43.3%), followed by car (26.7%) and on foot (20%); most injured people were drivers (60%). The number of people injuries as motor vehicle passenger was almost equal between males and females (5 and 4 respectively). Injuries occurred above all on roads outside the village (68.3%).

As mentioned above, traffic crashes were the primary cause of fatal injuries (n = 10). Two pedestrians were killed and eight motor-vehicle riders: five drivers (1 car, 2 motorcycle and 2 tractor drivers) and three car passengers. Three crashes involved more than one injured person with the following consequences: (1) the first had four injured people, three recovered and one died; (2) the second had two injured people, one recovered and one was disabled; (3) the last one had two injured people and both recovered completely.

#### Falls (35 injuries from 35 households)

Of 35 fall injuries, 28 were among males and 29 among adults – one in three (12 cases) were among people aged 65 years and above. Falls on the same level and from a roof were the most common kinds of falls reported (8 cases each), followed by fall from tree (n = 7) and from stairs (n = 5). Among older people, fall on the same level and from stairs were most common (4 cases each). Falls from trees occurred among adults and during work activities. This is common in rural Twiserkan, especially during the walnut harvest. Four deaths were fall-related: three occurred close to the injurious event (a man fell from a walnut tree, an older one fell from a roof, a woman aged 78 years on the stairs), and one some days later (a man aged 80 years following a pelvis fracture sustained after a fall when walking).

#### Burns (15 injuries from 8 households)

As many as 11 out of 15 burns were among males and 6 cases were paediatric burns (0–15 years). All burns occurred at home. Contact with flame was the most common cause (n = 12), several injurious events occurred when manipulating gas equipment used for cooking or heating. Other burns resulted from contact with hot liquids, steam or other gas (n = 3). Three events had more than one injured person: the first had three (two recovered and one died), the second had two (one recovered and the other one disabled) and the third one led to as many as five casualties.

Burns were the second cause of fatal injuries: six casualties resulting from two injurious events. The first occurred in the evening while the family members (5 persons) were sitting in a room and one of them was trying to unscrew a small gas capsule (picnic gas stove), to prevent a gas leak. According to the interviewee, the capsule came off suddenly, caught fire and all five persons received serious burn injuries. They were taken to the hospital but all five died after a few days. The second event occurred in the morning and involved three people. In circumstances similar to the preceding case – a gas leak from a gas capsule – the house caught fire suddenly. The three family members were also taken to the hospital but the family's one-year old child died after a few days.

### Fatal and non-fatal unintentional injury consequences

Table 2 shows the characteristics of consequences of the fatal and non-fatal unintentional injuries, all injuries aggregated and for three injury mechanisms: traffic, fall, and burn. Fracture was the most frequent nature of injury, encompassing about half of the all cases and 86% of fall-related injuries. The category "organ system injury/internal injury" refers to damage of some vital internal systems such as respiratory system, blood circulation system. In cases where more than one nature of injury was reported, focus was placed on the most serious one, according to WHO guidelines [29].

**Table 2:** Consequences of the fatal and non-fatal unintentional injuries by mechanism (June 1, 2005-May 31, 2006)

**Consequences**	**All ** **(n = 134)** **Number (%)**	**Traffic ** **(n = 60)** **Number (%)**	**Fall ** **(n = 35)** **Number (%)**	Burn* **(n = 15)** **Number**
**Physical nature**				
Fracture	66 (49.3)	32 (53.3)	30 (85.7)	-
Concussion	17 (12.7)	15 (25.0)	2 (5.7)	-
Cut, bite or other open wound	15 (11.2)	4 (6.7)	1 (2.9)	-
Burn	15 (11.2)	-	-	15
Poisoning	7 (5.2)	-	-	-
Organ system injury/internal injury	6 (4.5)	3 (5.0)	-	-
Bruise or superficial injury	4 (3.0)	4 (6.7)	-	-
Sprain/strain	3 (2.2)	1 (1.7)	2 (5.7)	-
Unspecified	1 (0.7)	1 (1.7)	-	-
				
**Body region**				
Head/face	19 (14.2)	17 (28.3)	1 (2.9)	1
Upper limb	18 (13.4)	5 (8.3)	8 (22.9)	-
Lower limb	27 (20.1)	15 (25.0)	7 (20.0)	1
Neck/shoulder/lower back/rib	10 (7.4)	4 (6.7)	3 (8.6)	2
Pelvis/hip	8 (6.0)	1 (1.7)	7 (20.0)	-
Internal system	7 (5.2)	-	-	-
Multiple regions	42 (31.3)	16 (26.7)	9 (25.7)	10
Unspecified	3 (2.2)	2 (3.3)	-	1
				
**Severity/recovery**				
Complete recovery	54 (40.3)	25 (41.7)	16 (45.7)	3
Partial recovery	31 (23.1)	14 (23.3)	10 (28.6)	2
Disability	22 (16.4)	11 (18.3)	5 (14.3)	4
Death	26 (19.4)	10 (16.7)	4 (11.4)	6

*As the number of burns amounted to 15, no percentages are presented.

For all injuries aggregated, the first most common injured single body region was the lower limb (20.1%), followed by the head/face (14.2%) and the upper limb (13.4% respectively). The most commonly injured single body region for traffic injuries was head/face (28.3%) followed by lower limb (25%) and for fall-related injuries was upper limb (22.9%) followed by lower limb and pelvis/hip equally (each 20%).

Whenever an injured person had more than one injured body region, it was considered and coded as "multiple regions"; which occurred in nearly one in three injuries (31.3%). As for the injury severity, as mentioned above, almost one in five injuries was fatal. Twice as many injured people recovered completely and 23.1% recovered partially.

### Interviewees' suggestions for prevention

Interviewees came up with quite a lot of suggestions concerning what more could be done to contribute to injury prevention and these suggestions varied considerably in kind depending on what actor they were asked to reflect upon: the authorities, the Behvarzes or the people themselves. The suggestions proposed were organized in a number of different categories and are presented in Table 3, shown in percentages both by number of suggestions and by number of households and also considering all injuries aggregated and traffic, fall and burn injuries separately.

**Table 3:** Household-based suggestions about activities that can be undertaken by various actors in order to control and prevent injuries

	**All**	**Traffic**	**Fall**	**Burn**
	no. household=117	no. household=56	no. household=35	no. household=8
**People's opinions/suggestions**	% suggestions *(% households)*	% suggestions *(% households)*	% suggestions *(% households)*	Number
**Authorities**				
Engineering/building (infrastructure/signalization/product)	31.1 (48.7)	37.4 (60.7)	34.8 (45.7)	1
Provision of daily services (accessibility/availability)	19.7 (30.8)	19.8 (32.1)	19.6 (25.7)	3
Financial support	12.0 (18.8)	7.7 (12.5)	23.9 (31.4)	-
Enforcement	10.4 (16.2)	12.1 (19.6)	6.5 (8.6)	1
Provision of emergency services (accessibility/availability)	8.7 (13.7)	5.5 (8.9)	6.5 (8.6)	5
Education	8.2 (12.8)	8.8 (14.3)	4.3 (5.7)	1
Maintenance/repair	7.7 (12.0)	8.8 (14.3)	2.2 (2.9)	1
Other	2.2 (3.4)	-	2.2 (2.9)	-
Total number of suggestions	183	91	46	12
				
**Behvarzes**				
Instruction/education/information	62.4 (58.1)	69.4 (60.7)	63.3 (54.3)	3
Post trauma care (health house/home visit)	16.5 (15.4)	14.3 (12.5)	13.3 (11.4)	1
Availability/accessibility of Behvarz all day round/drug and equipment of health house	10.1 (9.4)	10.2 (8.9)	10.0 (8.6)	1
As good as it can be	9.2 (8.5)	4.1 (3.6)	13.3 (11.4)	1
Other (Insist on of financial support by authorities and pass people's problem to them)	1.8 (1.7)	2.0 (1.8)	-	1
Total number of suggestions	109	49	30	7
				
**People**				
Behave in a safe manner	34.1 (37.6)	35 (37.5)	30.0 (34.3)	3
Cooperation together	18.6 (20.5)	13.3 (14.3)	20.0 (22.9)	3
Cooperation (with authorities)	17.8 (19.7)	20.0 (21.4)	20.0 (22.9)	1
Compliance	12.4 (13.7)	18.3 (19.6)	7.5 (8.6)	-
Engineering/building	8.5 (9.4)	3.3 (3.6)	17.5 (20.0)	1
Education (pay attention to planned education for them and/or pass to their children)	8.5 (9.4)	10.0 (10.7)	5 (5.7)	1
Total number of suggestions	129	60	40	9

#### Authorities

Almost one-third (31.1%) of the suggestions concerned changes in the living and commuting environment e.g., engineering and/or building (including infrastructure such as road construction and asphalting; signalization and product design). Proposals regarding the infrastructure and modernization of the traffic were most common, followed by suggestions concerning the provision of daily services (19.7 %), e.g., piped gas and even fire station for burns prevention, outdoor lighting for fall prevention and telephone for rapid contact with the health services at the time of injury event. People also mentioned the need for financial support for safety improvement in the village and even for better housing (12%). Law enforcement was also raised, in particular for traffic injury prevention (10.4%).

#### Behvarzes

For the Behvarzes, focus was placed largely on the provision of education to the people (about 60–69%), including various forms of continuous education in the population and informing upwards in the health system. They pointed to safety education in general and also to different aspects of safety educations such as road traffic safety education for the young (especially in relation to motorcycling) and home safety (targeting childcare and supervision). The latter was also considered as a way to promote the development of safe behaviour in children. The second important category of suggestions concerning the Behvarzes was about post trauma care, including both health house and home visits (16.5 %). Greater availability and accessibility to the Behvarzes and also to drugs and equipment (10.1%) came in third place.

#### People themselves

About one in three respondents considered that people themselves could behave in a safer manner to help control and prevent injuries. Thereafter, respondents raised the issue of cooperation between people (18.6%), including assisting each other in the event of injury, e.g., by transferring injured people to the health facilities. The third category of suggestions was about people's cooperation with the authorities to improve safety in the village-environment (17.8%). People also raised issues related to compliance, in-house engineering, and being good role-models for children, including teaching them safe behaviours and practices.

## Discussion

The study reveals that injuries among people in rural areas affect mainly males and also people of working age, which is in line with the injury distribution in other settings [2,5,9,11,14,17,30,31]. One finding is the relative importance of injuries on the road, not only because of their severity (10 out 26 deaths), but also because of their frequency. They are indeed as numerous as injuries in the home. Similar results were obtained in earlier studies from Vietnam [5] and Nicaragua [18].

Thus, traffic injuries are not only a concern among urban people in Iran but also among rural dwellers [32]. As motorized commuting is on the increase even in rural Iran, it is important to pay attention to road safety [4,32]. In particular, the safety of motorcyclists must be carefully considered as motorcycles are a popular means of transport [22] – as is the case in many Asian countries [7,34].

According to the opinions of the people interviewed – coming from households that have been affected by a severe RTI during the past year -, various things can be done: roads can be better designed and maintained, individual protection legislation (e.g. compulsory safety belt and helmet wearing) could be enforced, people could comply with and adopt safer behaviours, Behvahzes could educate the population and convey information of relevance for injury control and prevention upwards in the health system. Interestingly, many of those suggestions find an echo in the recommendations found in the WHO report on road traffic injuries, in particular concerning making road safety a political priority, enacting and enforcing legislation, managing infrastructure to promote safety for all, and campaigning for greater attention to road safety [4].

Injuries at home are also a concern in rural areas just as they are for instance in Pakistan, a neighbouring country, where they form the majority of injuries [9]. In the district studied herein, burns may require special attention, if not because of their frequency, because of their severity (see also earlier Iranian studies [35-38]). Burns also affect children to a greater extent, which is consistent with an earlier study from Bangladesh that showed that the incidence of burns among rural children was more than four times higher than among urban children [39].

In the Twiserkan district – and perhaps even in Iran as a whole – gas equipment used for cooking or heating may warrant special attention. Some villages still do not have gas mains and people use gas capsules and/or other heating equipment that is poorly adapted to in-house use. At present, fire stations are far from most villages and people did mention this as a matter of concern.

For its part, the prevention of falls is undermined by the vast number of different situations leading up to them, which is a challenge for community-based education programs. Potentially more severe falls from a height, e.g. from a roof and falls from a tree may constitute important targets. The latter occur during work activities, mainly during the walnut harvest. This work is done in a traditional, non-technical manner and every year some people fall from large trees and are injured or killed. The results of one study on safety assessment of agricultural machinery in Iran showed that in 60% of cases agricultural injuries were severe [40]. It ought to be emphasized that an important number of falls affect older people, which has been also observed in an earlier study showing that falls from standing height, falls during walking and falls on stairs were important risk factors for hip fracture for older patients [41]. Fall-related injury prevention may require not only environmental improvements in and around the house but also, in the long run, changes in health behaviours (e.g. eating, smoking, and exercising) so as to reduce individual susceptibility to fall and also recovery after fall.

Generally, people frequently mentioned that Behvarzes could play an important role in safety education matters on the local level. Behvarzes already have face-to-face meetings with community members as part of their traditional duties. Also, in recent years, the Ministry of Health and Medical Education has introduced a number of home safety program [20,35,42], and provided Behvarzes with educational packages, which is consistent with international literature.

To our knowledge, there is a dearth of studies conducted thus far in Iran or in other rural settings that have collected people's opinions and suggestions about injury control and prevention. This study shows that rural people have a lot of ideas which can be considered for the conception and implementation of context-relevant measures for injury prevention in their community. In particular, people from households where injuries have occurred during the past year consider that not only a change in their own behaviour but also environmental changes and the provision of information and education are needed. We hope that the suggestions highlighted, though not fully representative of the whole rural population, will be taken into account in future developments of safety measures and programs, in both the Twiserkan district and other districts.

Because of the routines in place [25] and the relatively small size of the catchment areas, we have good reasons to believe that the study offers an accurate coverage of the severe injuries incurred in the population under study during the study period. It is indeed very likely that health houses do have a complete coverage of injuries leading to hospitalization and death in their community [25]. In spite of the fact that collecting injury data was a relatively new procedure when the cases were identified, we regard the likelihood of missing cases as very unlikely given that the injuries covered are relatively severe, that the Behvarzes are well anchored in their community, and that those communities are relatively small.

Before concluding, it ought to be underlined that the study covers one district only and is limited to one year of observation. Because of this, it is not possible to extrapolate our results to any other time period or district. Yet, some results can be regarded as a matter for investigation in other districts as well (e.g., traffic related injuries or burns).

## Conclusion

Traffic injury is an important cause of severe and fatal injury among people from rural areas. Its prevention requires a variety of measures under the responsibility of different actors. Behvarzes may play an important role in both injury surveillance and in identifying context-relevant means of prevention.

## Competing interests

The authors declare that they have no competing interests.

## Authors' contributions

FR-S has made substantial contributions to the conception and design of the study, took responsibility for and coordinated the acquisition of data, which she analyzed. She took part actively in the analysis of the data and in the writing up of the manuscript. LL, MN and MS contributed to the conception and design of the study. LL and MN were closely involved in the data collection process and took active part in the data analysis, result interpretation and manuscript writing. MS contributed to the study design, data acquisition and results interpretation. All authors read and approved the final manuscript.

## Pre-publication history

The pre-publication history for this paper can be accessed here:


